# Study on the Chemical Characterization and Hypolipidemic Function of *Nelumbo nucifera* Based on Its Flavonoid Components

**DOI:** 10.3390/molecules29235798

**Published:** 2024-12-09

**Authors:** Leyi Yang, Chang Zhou, Rong Huang, Yuan Cai, Bin Liu, Mimi Yu, Yanyan Jiang

**Affiliations:** 1 School of Chinese Materia Medica, Beijing University of Chinese Medicine, Beijing 102488, China; 18811305627@163.com (L.Y.); zhouchang0226@163.com (C.Z.); ht15286223413@163.com (R.H.); cai13263208090@163.com (Y.C.); 2 Beijing Institute for Drug Control, NMPA Key Laboratory for Quality Evaluation of Traditional Chinese Medicine (Traditional Chinese Patent Medicine), Beijing Key Laboratory of Analysis and Evaluation on Chinese Medicine, Beijing 102206, China; 3 The Key Research Laboratory of “Exploring Effective Substance in Classic and Famous Prescriptions of Traditional Chinese Medicine”, The State Administration of Traditional Chinese Medicine of the People’s Republic of China, Beijing 102488, China

**Keywords:** *Nelumbo nucifera*, quantitative fingerprint, functional foods, hyperlipidemia

## Abstract

*Nelumbo nucifera* has great value and development prospects in hypolipidemic applications. In this study, we comprehensively screened out multi–index components relevant to the quality of *N. nucifera* based on the hypolipidemic function of the flavonoid fraction of *N. nucifera* (FFN) combined with chemical characterizations. Firstly, in vitro antioxidant and cell experiments evaluated the hypolipidemic function of the FFN. Secondly, the chemical compositions of *N. nucifera* were identified by UPLS–MS^n^ technology. Then, the multi–index flavonoid components (rutin, hyperoside, isoquercitrin, quercetin–3–O–*β*–D–glucuronide, astragalin, and quercetin) were determined using a quantitative fingerprint combined with multivariate statistical data analysis. Finally, the quality of *N. nucifera* was scientifically evaluated by multi–index quantitative analysis combined with multivariate statistical data analysis, which was used to study the relationship between the content of flavonoid components and the overall quality. The above–mentioned research lays a material foundation for improving the quality standards of *N. nucifera*, providing a basis for developing functional foods to improve dyslipidemia.

## 1. Introduction

*Nelumbo nucifera* (Gaertn), belonging to the genus *Nelumbo* in the Nymphaeaceae family, is a homologous medicinal plant and food recorded in Chinese Pharmacopeia, version 2020 [1]. With the continuous development of society, the prevalence of glycolipid metabolic diseases such as obesity, fatty liver, and hyperlipidemia has shown a rapid upward trend in the world, seriously threatening people’s health [2,3]. In a diet–based regimen, functional foods as a means of treating diseases have received significant attention [4]. *Nelumbo nucifera*, which is widely used in preventing or managing chronic metabolic diseases such as hyperlipidemia, has typically been used in the development of nutraceuticals and functional foods [5,6]. In addition, according to the data regarding all the healthy food prescriptions on the official website of the State Food and Drug Administration—the domestic health food database—there are 258 kinds of health foods containing *N. nucifera*, and they are mainly used for reducing weight, regulating blood lipids, and relaxing the bowels [7]. Studies on bioactive components have shown that chemical constituents are responsible for the function of natural products, while flavonoids are one of the main active ingredients in *N. nucifera* [8,9]. Flavonoids are bioactive polyphenolic phytochemicals found in natural foods (soybean, celery, tangerine peel, etc.) and have attracted wide attention due to their antioxidant properties [10,11]. In addition, flavonoids also have multiple biological effects, such as anti–inflammatory effects, antibacterial effects, protection of cardiovascular and cerebrovascular systems, hypolipidemic effects, hypoglycemic effects, etc. [12]. Our previous study showed that the two flavonoids in *N. nucifera*, hyperoside and quercetin–3–O–*β*–D–glucuronide, displayed advantageous hypolipidemic activity [13]. In our previous research, the preparation method of FFN was established. On this basis, in order to provide a pharmacological basis for establishing a quality control method for lotus leaf flavonoids, we decided to explore and confirm the hypolipidemic effects of FFN.

Usually, in herbs of a natural origin, there are many factors, such as the species, cultivation area conditions, harvesting, processing, transportation and storage conditions, extraction, purification, ADME (absorption, distribution, metabolism, and excretion), and interaction of diverse components, that can affect the quality of TCM [14], which renders the study of TCM quality a systematic endeavor. Therefore, to ensure the quality of herbs, it is important to control their chemical constituent contents. Currently, in terms of the quality standards of *N. nucifera*, the Chinese Pharmacopoeia (Part 1, version 2020) takes nuciferine as the index component for content determination, and the quality control of *N. nucifera* is mainly based on its alkaloid contents. But the therapeutic effects of Chinese medicine are often the result of the synergistic action of multiple components [15]. Determining the content of a single compound or a class of compounds alone is insufficient to fully reflect the quality of Chinese medicine. Our previous research showed a huge difference in the types and contents of the functional components in *N. nucifera*, which are closely related to their activities [16]. Therefore, it is necessary to carry out quantitative research on flavonoid components to comprehensively evaluate the quality of *N. nucifera*.

**Figure 1:**
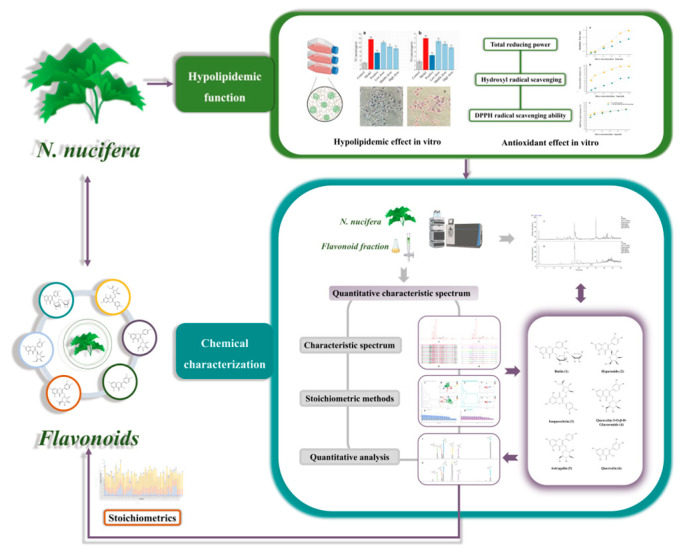
Work flowchart.

In this study, a trio of pharmacological experiments, chemical characterizations, and chromatographic fingerprints were combined to comprehensively screen and limit the multi–index components relevant to the quality of *N. nucifera* and FFN. Firstly, the antioxidant capacity of FFN was determined, and a hyperlipemia model induced by sodium oleate in HepG2 cells was established to evaluate the hypolipidemic activity of the FFN. Secondly, a rapid and sensitive UHPLC–Q–TOF–MS^n^ technology was established for comprehensively analyzing chemical constituents in *N. nucifera*. Ultimately, chromatographic fingerprints combined with chemometrics were used to identify and screen the multi–index components. The quantitative fingerprint of a traditional Chinese medicine can reflect the characteristics of its chemical types and its composition by finding the commonness of chemical components of the same traditional Chinese medicine group, which has both specificity and characteristics [17]. Our work analyzed the quantitative fingerprint information of 51 batches of *N. nucifera* and 10 batches of FFN. The multi–index components of *N. nucifera* (rutin, hyperoside, isoquercitrin, quercetin–3–O–*β*–D–glucuronide, astragalin, and quercetin) were screened out combined with similarity analysis (SA), hierarchical cluster analysis (HCA), principal component analysis (PCA), and orthogonal partial least squares discriminant analysis (OPLS–DA). Then, a content determination method of multi–index components was established, and the relationship between the total content of 6 different components and the quality of 51 batches of *N. nucifera* was analyzed by PCA, CA, and HCA to improve the quality standards of *N. nucifera* and ensure the nutritional value of *N. nucifera*. The above research improves the quality standard of *N. nucifera*, controls and evaluates the raw materials of health food, and provides a reference for the further development and research of lipid–lowering health care products. The workflow of this study is shown in Figure 1.

## 2. Results and Discussion

### 2.1. Results of UHPLC–Q–TOF–MS^n^ Analysis

**Figure 2:**
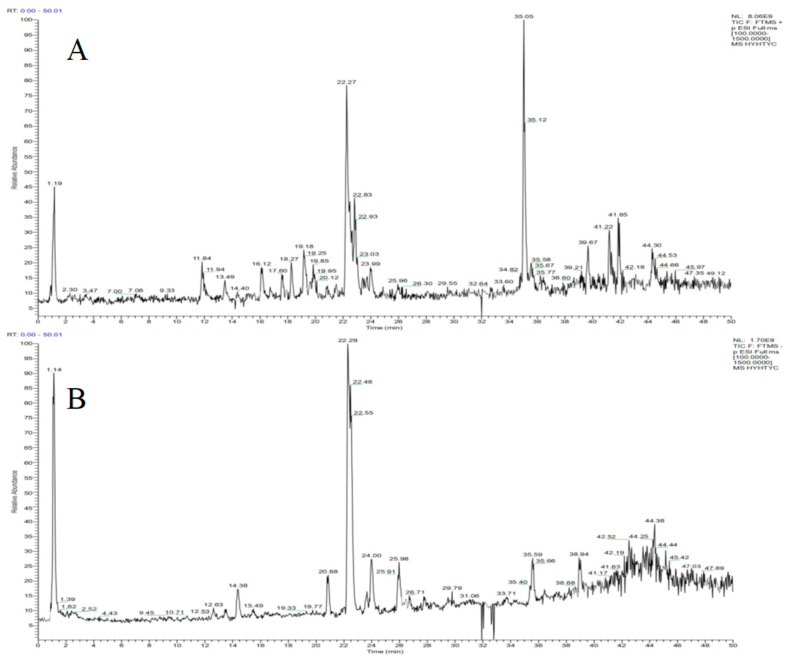
The total ion chromatograms of *N. nucifera*: (**A**) positive ion mode; (**B**) negative ion mode.

The chemical components of *N. nucifera* herb were fully detected under ideal UHPLC–Q–TOF–MS^n^ conditions. A total of 105 compounds (45 flavonoids, 22 alkaloids, 10 amino acids, 18 organic acids, and 10 other compounds) were identified using chromatographic retention durations, MS/MS data or literature, and elemental composition analysis within an error of 5 ppm (Figure 2; Table S1).

### 2.2. Antioxidant Effect Evaluation In Vitro

#### 2.2.1. Determination of Total Reducing Power of FFN

**Figure 3:**
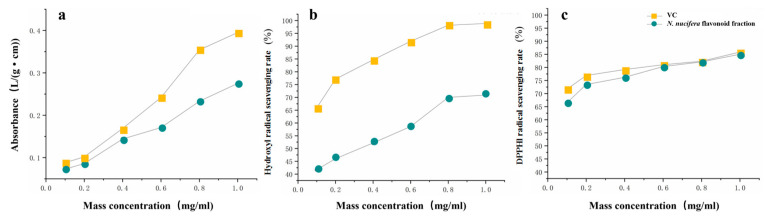
Results of antioxidant effect evaluation in vitro: (**a**) total reducing power of FFN and Vc; (**b**) hydroxyl radical scavenging ability of FFN and Vc; (**c**) DPPH radical scavenging ability of FFN and Vc.

Potassium ferricyanide [K_3_Fe (CN)_6_] can be reduced to potassium ferricyanide [K_4_Fe (CN)_6_] using reducing agents. Under acidic conditions, potassium ferricyanide [K_4_Fe (CN)_6_] can react with Fe^3+^ to form produce Prussian blue, which has a substantial absorption at a wavelength of 700 nm, and the higher the absorbance, the greater the reduction ability [18]. The figure shows that, within a specific concentration range, the total reducing capacity of Vc (Vitamin C) and FFN increased with the increase in concentration, indicating that both of them can inhibit the transfer of electrons by free radicals and terminate the free radical chain reaction (Figure 3a). However, compared to previous studies, the difference in overall reduction power of FFN and Vc was greater, which could be attributed to the extraction method [19,20].

#### 2.2.2. Determination of Hydroxyl Radical Scavenging Ability of FFN

Fe^2+^ can combine with hydrogen peroxide to form hydroxyl radicals, which are highly active but have a very short survival time. Salicylic acid can trap hydroxyl radicals, resulting in colorful compounds with significant absorption at 510 nm. Antioxidants can compete with salicylic acid for hydroxyl radicals, reducing the synthesis of colored compounds. As a result, the hydroxyl radical scavenging ability of the tested compounds can be determined by measuring the absorbance of the solution at 510 nm. Figure 3b shows that the scavenging ability of both Vc and FFN on hydroxyl radicals increased with concentration. In the concentration range of 0.1~1.0 mg/mL, FFN’s scavenging rate increased from 41.734% to 70.895%. The results show that, at the same concentration, FFN has a scavenging effect on hydroxyl radicals. However, the scavenging ability is not as good as Vc (Figure 3b), which is consistent with previous research findings on the hydroxyl radical scavenging ability of flavonoids in *N. nucifera* [21].

#### 2.2.3. Determination of DPPH Radical Scavenging Ability of FFN

The DPPH radical method can be used to test antioxidant capacity, and it has the advantages of being simple to use, easy to detect, and inexpensive. The DPPH radical is a stable free radical centered on nitrogen, having arc pair electrons, a deep purple color, and a prominent absorption peak at 517 nm. When antioxidant–active chemicals are present in the reaction system, the arc pair electrons will be paired, resulting in a pale yellow or colorless solution. The clearance rate can be estimated by evaluating the degree of color fading to determine the tested substance's ability to scavenge DPPH radicals [22]. Figure 3c shows that, within a specific concentration range, the scavenging ability of Vc and FFN on DPPH radicals increases with the increase in concentration, and the concentration range is 0.1–1.0 mg/mL. The DPPH radical scavenging rate of FFN increased from 66.478% to 85.039%. When the mass concentration was lower than 0.6 mg/mL, the scavenging rate DPPH radical scavenging rate of FFN was lower than that of Vc. However, when the concentration gradually increased to 1.0 mg/mL, there was almost no difference in the DPPH radical scavenging rate between Vc and FFN. The evidence shows that high concentrations of FFN demonstrate a strong ability to scavenge DPPH radicals (Figure 3c).

### 2.3. Evaluation of Hypolipidemic Activity

#### 2.3.1. In Vitro Cytotoxicity

To explicate the toxicity of sodium oleate and FFN, the cell viability in HepG2 cells was measured by the MTT assay. HepG2 cells were treated with sodium oleate (10–100 µg/mL), FFN (25–600 µg/mL), and simvastatin (3–100 µM) for 24 h. The control (HepG2 cells lacking both sodium oleate and FFN) showed cell viability exceeding 90%, suggesting non-cytotoxicity (Figure S1) [23]. Therefore, FFN (25, 50, 100, 200 µg/mL) and simvastatin (3, 10, 30 µM) were used to treat the hyperlipidemia model in HepG2 cells which were induced by sodium oleate.

#### 2.3.2. Sodium Oleate–Induced TG Accumulation in HepG2 Cells

Sodium oleate significantly increased cellular TG content in HepG2 cells in a concentration-dependent manner, as compared to controls lacking sodium oleate (Figure S2). Therefore, to establish the hyperlipidemia cell model triumphantly, the consistency of 60 µg/mL of sodium oleate was selected to induce the cells. After staining with oil red O staining solution, the intracellular lipid droplets were dyed orange. There were only a few lipid droplets in HepG2 cells in the blank group. The lipid droplets in high-fat HepG2 cells induced by sodium oleate increased significantly, which was consistent with the results of increased intracellular TG content (Figure S3).

#### 2.3.3. The Effect of FFN on TC and TG Accumulation in Sodium Oleate–Induced HepG2 Cell Hyperlipemia Model

**Figure 4:**
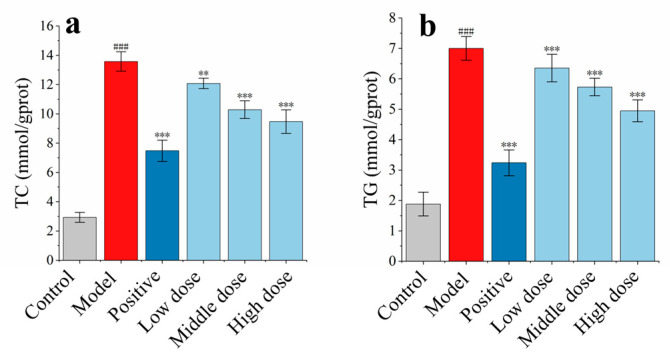
The effect of FFN on TC and TG contents in HepG2 cells induced by sodium oleate (*n* = 6): (**a**) the effect of FFN on TC; (**b**) the effect of FFN on TG. Compared with blank group, *^###^ p* < 0.001; compared with model group, ^**^ p < 0.01, ^***^ p < 0.001.

The MTT findings were used to determine the right dosage for each group. FFN was administered at 50, 100, and 200 µg/mL for low, medium, and high dosages, respectively. The positive control group has a simvastatin concentration of 30 µM. SPSS (version 22.0) software was used for statistical analysis of the experimental data. HepG2 cells were successfully stimulated by sodium oleate to create a high-lipid model, as evidenced by the considerable differences in TC and TG between the model group and the blank group. The TC and TG of the positive control group were considerably different from those of the model group, demonstrating the viability of the experimental approach. In comparison to the model group, there was a substantial decrease in the TC content (*p* < 0.01, *p* < 0.001, *p* < 0.001) and TG content (*p* < 0.001, *p* < 0.001, *p* < 0.001) in the low-, medium-, and high-dose groups of FFN (Figure 4). In summary, there is clear hypolipidemic activity in the low-, medium-, and high–dose groups of FFN.

### 2.4. Quantitative Fingerprint Analysis

#### 2.4.1. Method Validation of Fingerprint and Similarity Analysis

The method of the fingerprints of *N. nucifera* and FFN was validated in terms of precision, repeatability, and stability. Peak 8 (quercetin–3–O–*β*–D–glucuronide) was selected as the reference peak which had better separation and intermediate retention time. In the precision investigation, the relative standard deviation (RSD) of relative peak areas was 0.036–0.206%, and the RSD of relative retention time was 0.056–2.695%; all of these values were less than 3.0%, indicating that the precision of the instrument met the requirements. In the repeatability investigation, the RSD of relative peak areas was 0.136–0.544%, and the RSD of relative retention time was 1.038–2.670%; all of these values were less than 3.0%, indicating that the repeatability of the method met the requirements. In the stability investigation, the RSD of relative peak areas was 0.064–0.325%, and the RSD of relative retention time was 0.069–2.815%; all of these values were less than 3.0%, indicating that the sample solution was stable within 24 h.

**Figure 5:**
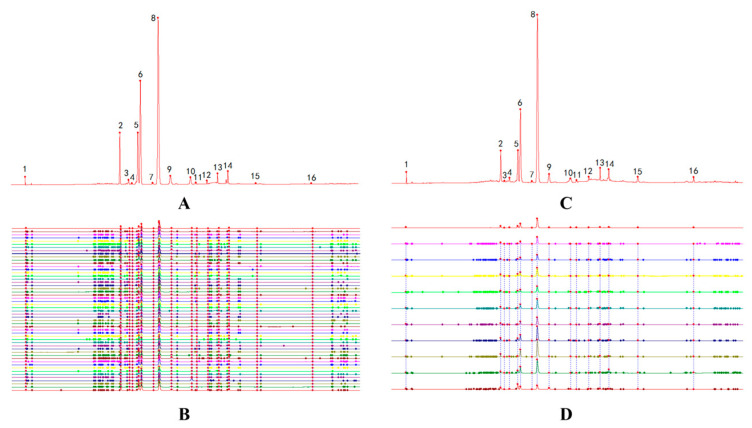
The reference chromatogram and fingerprints of *N. nucifera* and FFN (rutin (peak 3), hyperoside (peak 5), isoquercitrin (peak 6), quercetin–3–O–*β*–D–glucuronide (peak 8), astragalin (peak 9), quercetin (peak 14), and kaempferol (peak 15)): (**A**) the reference chromatogram of *N. nucifera*; (**B**) the fingerprints of *N. nucifera*; (**C**) the reference chromatogram of FFN; (**D**) the fingerprints of FFN.

*N. nucifera* samples (51) and FFN (10) samples were prepared, and fingerprint analysis was carried out according to chromatographic conditions. Data were imported into the “Chinese Medicine Chromatographic Fingerprint Similarity Evaluation System software” (version 2012). The chromatographic diagram of sample S1 was used as the reference atlas, the median method was adopted, and Marker peaks were matched through multi–point correction to establish the superposition atlas and the control atlas (Figure 5A–D). Sixteen common peaks in HPLC fingerprints were screened out, and seven flavonoid components were identified as rutin (peak 3), hyperoside (peak 5), isoquercitrin (peak 6), quercetin–3–O–*β*–D–glucuronide (peak 8), astragalin (peak 9), quercetin (peak 14), and kaempferol (peak 15) by comparing the PDA spectrogram and the retention time with that of the reference substance. The similarity between the samples of 51 batches of *N. nucifera* was 0.958~1.000 (Table S4), and the similarity between the samples of 10 batches of FFN was 0.796~0.999 (Table S5). However, the overall similarity between the samples of 10 batches of FFN was higher than that of 51 batches of *N. nucifera*, which indicates that the quality of purified *N. nucifera* is more stable.

#### 2.4.2. Multivariate Statistical Data Analysis of Fingerprints

**Figure 6:**
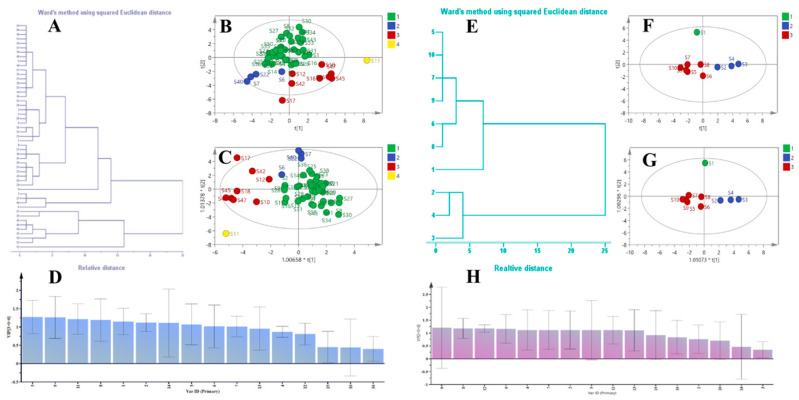
Results of HCA, PCA, and OPLS–DA analysis of *N. nucifera* and FFN: (**A**) HCA tree diagram of *N. nucifera*; (**B**) PCA scatter diagram of *N. nucifera*; (**C**) OPLS–DA scatter diagram of *N. nucifera*; (**D**) VIP value of common peaks in *N. nucifera;* (**E**) HCA tree diagram of *FFN*; (**F**) PCA scatter diagram of FFN; (**G**) OPLS–DA scatter diagram of FFN; (**H**) VIP value of common peaks in FFN.

The HCA results showed that 51 batches of *N. nucifera* samples could be divided into four categories. The first category is S1–S5, S8, S9, S13 to S16, S19 to S21, S23 to S39, S41, S43, S44, and S48–S51, which are produced in AH, HB, HB, HN, JL, JS, JX, SD, SC, and ZJ. The second category is S6, S7, S22, and S40, which are produced in AH, JS, and SD. The third category is S10, S12, S17, S18, S42, and S45–S47, which are produced in the HB, HN, SD, and SC regions, and the fourth category is S11, which is from the HB region (Figure 6A). The results of PCA showed that 51 batches of *N. nucifera* can be divided into four categories, which is consistent with the HCA results (Figure 6B). The HCA and PCA results suggest that differences in *N. nucifera* components are likely to be related to environmental factors such as climate and soil at the time of cultivation and may also be related to the conditions under which they are processed, stored, and transported. Fifty-one batches of *N. nucifera* were analyzed by OPLS–DA and could be roughly divided into four categories (Figure 6C). Within a confidence interval of 0.95, chromatographic peaks with VIP > 1.0 were selected as difference markers. A total of 10 components with great influence on classification were screened, namely peaks 5, 9, 11, 8, 1, 2, 14, 3, 6, and 7 (Figure 6D).

The HCA results showed that 10 batches of FFN samples could be divided into three categories. The first category was S1, the second category was S2–S4, and the third category was S5–S10 (Figure 6E). In the unsupervised grouping mode, 10 batches of FFN can be divided into three categories, which is consistent with the HCA results (Figure 6F). Ten batches of flavonoid fractions were analyzed by OPLS–DA, which could be roughly divided into three categories (Figure 6G). Within a confidence interval of 0.95, chromatographic peaks with VIP > 1.0 were selected as difference markers. A total of 10 components with great influence on classification were screened, namely peaks 8, 9, 12, 6, 4, 7, 2, 5, 11, and 13 (Figure 6H).

**Figure 7:**
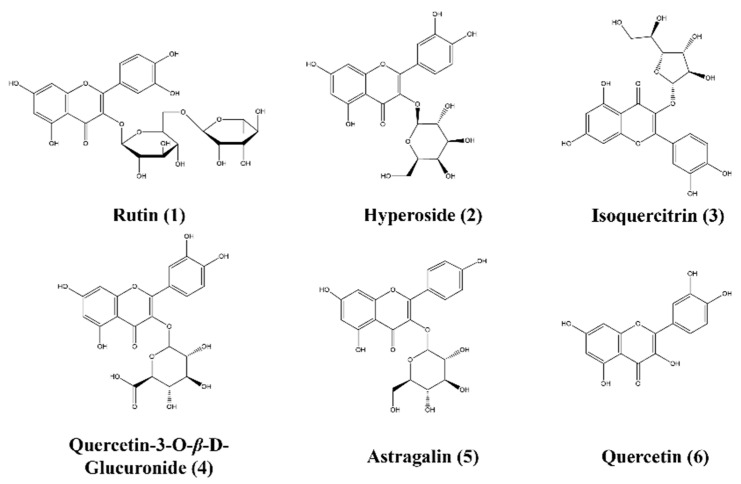
The chemical structure of six flavonoid multi–index components.

To synthesize the results of multivariate statistical data analysis of 51 batches of *N. nucifera* samples and 10 batches of FFN samples, we selected rutin (peak 3), hyperoside (peak 5), isoquercitrin (peak 6), quercetin–3–O–*β*–D–glucuronide (peak 8), astragalin (peak 9), and quercetin (peak 14) as markers of flavonoid quality differences in *N. nucifera* based on the screening results of the differential components of *N. nucifera* and FFN combined with in vitro chemical composition characterization (Table 1; Figure 7).

#### 2.4.3. Quantitative Analysis of Six Multi–Index Components

##### Method Validation of Quantitative Analysis

**Figure 8:**
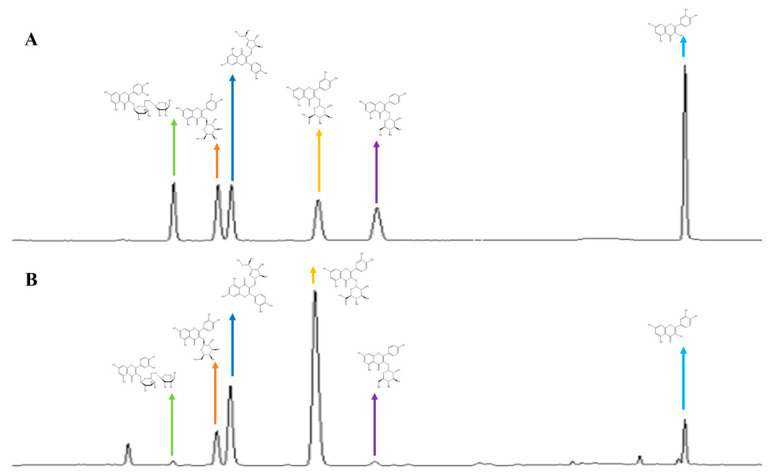
The chromatogram of multi–index components of *N. nucifera:* (**A**) the chromatogram of the reference solution; (**B**) the chromatogram of the sample solution.

A series of method validations of quantitative analyses were performed, and all met the requirements (Table 2; Figure 8). The correlation coefficient (R2) of each compound was higher than 0.999, which showed good linearity. The LOQ and LOD results indicated that the HPLC method was sensitive for quantitation. The RSD values of precision were less than 1.0%, indicating that the precision of the instrument met the requirements. The stability of the method was acceptable, with an RSD value within 3.0%, indicating that the sample solution was stable for 24 h. The durability of the method was determined by the same sample solution with three different columns: an Agilent Eclipse XDB–C18 column (4.6 mm × 150 mm, 5 μm), a Waters SunFire C18 column (5 μm, 4.6 mm × 250 nm), and an Agela Technologies Promosil C18 column (4.6 mm × 150 mm, 5 μm). The RSD values were all less than 3.0%, indicating that the durability of the method met the requirements. The repeatability met the requirements with an RSD within 3.0%. The recovery rates of each compound were in the range of 95.0–105.0%, and their RSD values were less than 3.0%. Therefore, the HPLC method was precise enough for the simultaneous quantitative evaluation.

##### Content Determination

**Figure 9:**
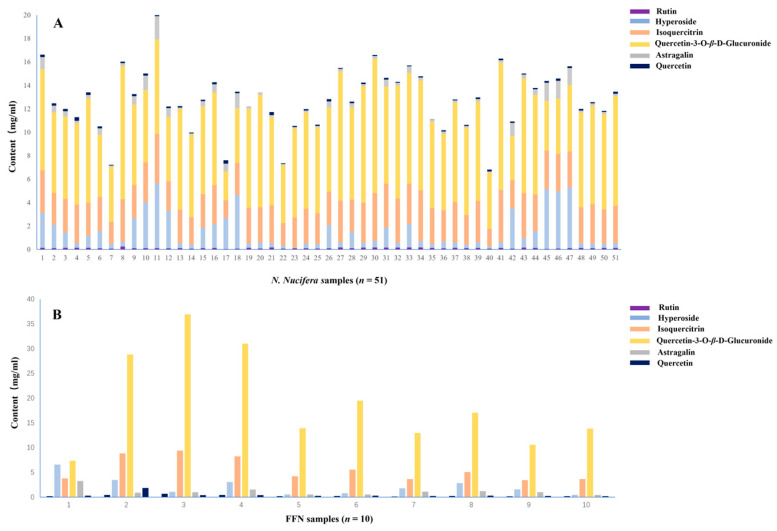
The contents of 6 flavonoids in *N. nucifera* and FFN (*n* = 51) and FFN (*n* = 10): (**A**) the contents of 6 flavonoids in *N. nucifera*; (**B**) the contents of 6 flavonoids in FFN.

Test solutions of *N. nucifera* samples (*n* = 51) and FFN (*n* = 10) were prepared and analyzed according to the chromatographic conditions for the determination of differential components. Among the *N. nucifera* samples (*n* = 51), the contents of rutin, hyperoside, isoquercitrin, quercetin–3–O–*β*–D–glucuronide, astragalin, and quercetin were distributed in the ranges of 0.0335–0.2469%, 0.0346–5.5595%, 1.5059–4.4790%, 2.4584–11.4653%, 0.0855–1.9885%, and 0.0109–0.3942% (Figure 9A, Table S6). Among the FFN samples (*n* = 10), the contents of rutin, hyperoside, isoquercitrin, quercetin–3–O–*β*–D–glucuronide, astragalin, and quercetin were distributed in the ranges of 0.1141–0.6765%, 0.4618–6.5770%, 3.4379–9.3935%, 7.3575–36.9243%, 0.4321–3.2773%, and 0.1804 9–1.8320% (Figure 9B, Table S7).

##### Multivariate Statistical Data Analysis

The relationship between the quality of *N. nucifera* and the total content of six flavonoid multi–index components was analyzed by PCA, CA, and HCA, and a limit of the total content of six flavonoid multi–index components in *N. nucifera* was set. In PCA, two PCs, with a cumulative variance contribution rate of 83.478%, were selected. Among them, the contribution of PC Z1 was 50.540%, which was much greater than that of PC Z2, indicating that PC Z1 was the leading indicator in the quality evaluation of *N. nucifera* (Table S1; Figure S4).

The initial factors loading matrix showed that rutin, hyperoside, quercetin–3–O–*β*–D–glucuronide, and astragalin have large load values (0.753, −0.800, 0.884, and −0.755) in PC Z1, proving that they are important for the quality evaluation of *N. nucifera*. In addition, isoquercitrin had the largest load value (0.811) in PC Z2, indicating that PC Z2 mainly reflected the content of isoquercitrin (Table S9). Based on the contribution of PC Z1 and Z2, we calculated the comprehensive score of each batch (Z) by the formula Z = 0.54540Z1 + 0.32938Z2 (Table S10). Then, a correlational calculation between the total content of six flavonoids in *N. nucifera* and their comprehensive score was carried out, and the correlation coefficient was equal to 0.859, indicating that the correlation was significant between them (Table S11). The above results proved that the total content of six flavonoids could evaluate the quality of *N. nucifera* comprehensively.

HCA with the total content of six flavonoids in 51 batches of *N. nucifera* samples was carried out. When the classification distance was set at 10, 51 batches of *N. nucifera* were divided into three categories. Among them, S5–S9, S15–S16, S20–S27, S29–S31, S35–S37, S39, S41–S48, and S50–S51 were clustered into the first category; S1–S4, S10–S11, S13–S14, S17–S18, S28, S32, S34, S38, and S40 were clustered into the second category; S12, S19, S33, and S49 were clustered into the last category. In the first category, the total content of five alkaloid Q–Markers was less than 0.30%; in the second category, the total content of five alkaloid Q–Markers was in the range of 0.30–0.90%; in the last category, the total content of five alkaloid Q–Markers was more than 0.90% (Figure S5).

##### Analysis of Quercetin–3–O–β–D–Glucuronide in *N. nucifera*

According to the PCA results, the correlation coefficient between quercetin–3–O–*β*–D–glucuronide and the first principal component was the largest, indicating that the contribution of quercetin–3–O–*β*–D–glucuronide in the total content of six flavonoids in *N. nucifera* could not be ignored. Therefore, it is necessary to study the effect of quercetin 3–O–*β*–D–glucuronide content on the quality of *N. nucifera*. The ratio of quercetin–3–O–*β*–D–glucuronide content to the total content of six different components was used as the index for cluster analysis, which showed that when the group distance was 5, 51 batches of commercially available *N. nucifera* could be divided into four categories: the first category included S17, S18, S42, and S45–S47; the second category included S10~S12; the third category included S1~S4, S6, S9, S15, S16, S26, S28, S31, S33, and S44. The fourth category included S5, S7, S8, S13, S14, S19~S25, S27, S29, S30, S32, S34~S41, S43, and S48~S51. Among them, the ratio of the first type is less than 38%, the ratio of the second type is between 38 and 50%, the ratio of the third type is between 50 and 63%, and the ratio of the fourth type is greater than 63%. The above results showed that the ratio of quercetin 3–O–*β*–D–glucuronide content to the total content of six different components in 82.35% (42 batches) of commercially available *N. nucifera* was more than 50%, indicating that quercetin–3–O–*β*–D–glucuronide content was higher in more than half of *N. nucifera*, which had a certain impact on the quality of *N. nucifera* (Table S12; Figure S6).

## 3. Materials and Methods

### 3.1. Plant Material

Fifty–one batches (S1–S51) of *N. nucifera* were obtained from multiple provinces in China (Table S11). Ten batches of *N. nucifera* used to prepare FFN were purchased from various provinces in China (Table S12). They were identified as the dried leaves of *N. nucifera* in the Nymphaeaceae family by Prof. Yuan Zhang (Beijing University of Traditional Chinese Medicine, Beijing, China).

### 3.2. Chemicals and Reagents

HPLC acetonitrile, MS acetonitrile, penicillin–streptomycin, 0.25% trypsin EDTA, and PBS buffer were purchased from Thermo Fisher Scientific Inc. (Waltham, MA, USA). AB–8 Macroporous Resin was purchased from Keda Resin Technology Co., Ltd. (Tianjin, China). Quercetin, hyperoside, isoquercitrin, rutin, kaempferol, and isorhamnetin were all purchased from the China Institute for Food and Drug Control (Beijing, China). Astragalin was purchased from Yuanye Bio–technology Co., Ltd. (Shanghai, China). Quercetin–3–O–*β*–D–glucuronide was purchased from Durst Biotechnology Co., Ltd. (Chengdu, China). Sodium hydrogen phosphate dodecahydrate was purchased from Sinopharm Group Chemical Reagent Co., Ltd. (Beijing, China). Sodium dihydrogen phosphate dihydrate was purchased from Hongxing Chemical Plant (Beijing, China). Trichloroacetic acid and formic acid were purchased from Fuchen Chemical Reagent Co., Ltd. (Tianjin, China). Ferric chloride was purchased from Yungtay Chemical Reagent Co., Ltd. (Tianjin, China). Methanol, absolute ethanol, and salicylic acid were purchased from Zhiyuan Chemical Reagent Co., Ltd. (Tianjin, China). Potassium ferricyanide was purchased from Beijing Chemical Plant. Vc was purchased from Huazhong Pharmaceutical Co., Ltd. (Hubei, China). Hydrogen peroxide solution was purchased from Jianning Pharmaceutical Co., Ltd. (Jianning, China). Ferrous sulfate heptahydrate was purchased from Bidd Medical Technology Co., Ltd. (Shanghai, China). RPMI 1640 medium, dimethyl sulfoxide (DMSO), BCA protein concentration determination kit, and oil red O dye solution were all purchased from Solaibao Technology Co., Ltd. (Beijing, China). A total cholesterol (TC) kit, triglyceride (TG) kit, and DPPH free radical scavenging ability kit were purchased from Jiancheng Institute of Biological Engineering (Nanjing, China). Fetal bovine serum (FBS) was purchased from Sijiqing Bioengineering Co., Ltd. (Hangzhou, China). Thiazole Blue (MTT), sodium oleate (SO), and Triton X–100 lysate were purchased from Yuanye Biotechnology Co., Ltd. (Shanghai, China). Simvastatin was purchased from Euitech Pharmaceutical Co., Ltd. (Shijiazhuang, China). Human hepatoma cell line HepG2 was purchased from the National Experimental Cell Resource Sharing Platform (Beijing, China).

### 3.3. Sample and Standard Preparation

*N. nucifera* was mixed with 200–fold methanol for an ultrasound for 40 min and then filtered, and 25 mL of the filtrate was precisely measured and then evaporated. The residue was dissolved into a 5 mL volumetric bottle with methanol and filtered by a 0.45 μm microporous filter membrane for UPLC and HPLC analysis of *N. nucifera*.

*N. nucifera* was mixed with 30–fold 70% ethanol for refluxing two times, 1.5 h each time, and then the extraction was filtrated, evaporated, and dried. The dried powder was dissolved with 70% ethanol (14 mg/mL); then, the AB–8 macroporous resin column was used, 3BV was removed by water, 3BV was eluted with 70% ethanol, the ethanol elution was collected, and the FFN was obtained by evaporation and drying.

The FFN (15 mg) was mixed with 5 ml of 70% methanol for a 20 min ultrasound and filtered by a 0.45 μm microporous filter membrane for UPLC and HPLC analysis. The FFN (2, 5, 10, 15, 20, and 25 mg) were mixed with 25 mL of 70% methanol for ultrasound for 20 min to study the antioxidant effect.

The FFN extraction was dissolved by DMSO and treated with various concentrations (0.1% DMSO) by a cell culture medium for Rcell experiments.

The appropriate amounts of kaempferol, isorhamnetin, rutin, isoquercitrin, quercetin, quercetin–3–O–*β*–D–glucuronide, and astragalin standard substances were accurately weighed, and methanol was added separately to prepare the reference solutions with the concentrations of 0.236 (A), 0.227 (B), 0.206 (C), 0.224 (D), 0.223 (E), 0.265 (F), 0.253 (G), and 0.264 (H) mg/mL.

The reference solution (A–H) was absorbed in 1 ml and dissolved with 10 mL methanol, a mixed reference solution. The above solutions were filtered using 0.45 µm microporous membranes before UPLC and HPLC analysis.

Vc (2, 5, 10, 15, 20, 25 mg) was mixed with 25 mL of 70% methanol for ultrasound for 20 min to form a positive control solution for studying the antioxidant effect.

### 3.4. UHPLC–Q–TOF–MS^n^ Analysis

#### 3.4.1. Chromatographic and Mass Spectrometry Conditions

UPLC analysis was performed on an ACQUITY^TM^ UPLC system (Waters, Milford, MA, USA). Samples were separated on a WATERS ACQUITY UPLCTM BEH C18 (50 mm × 2.1 mm, 1.7 µm) at 30 °C. The mobile phase consisted of methanol (A) and 0.2% formic acid aqueous solution (B), and the elution gradient was set as follows: 0–3 min, 3% A; 3–10 min, 3–15% A; 10–40 min, 15–100% A. The operating parameters were set as follows: the flow rate was 0.3 mL/min; the detection wavelength was 280 nm; the injection volume was 3 µL. Ultra–performance liquid chromatography with quadrupole Exactive plus Orbitrap mass spectrometry (UPLC–MS/MS) was used to identify the chemical compositions. The operating parameters of MS/MS were set as follows: ESI ion source; sheath gas and auxiliary gas: both nitrogen, purity > 99%; collision gas is helium, purity > 99.99%; sheath gas flow rate 40 arb, auxiliary gas flow rate 20 arb; ionization source voltage 4 KV; ionization source voltage 25 V; tube lens voltage 110 V. The sample was fully scanned by FT, with a mass spectrometry scan range of *m*/*z* 100–1500, and the detection resolution of the secondary mass spectra was scanned by a data–dependent scan (DDS). The three ions with the highest abundance in the primary mass spectrum were scanned for collision–induced dissociation (CID) fragmentation.

#### 3.4.2. Identification of Compounds

The compounds represented by peaks in the mass spectrum were identified by Xcalibur and Compound Discoverer (version 3.0).

### 3.5. Evaluation of Antioxidant Effect In Vitro

#### 3.5.1. Determination of Total Reducing Power

First, 0.2 mL sample solution with different concentrations of 2.3 mL of 0.2 mol/L phosphate buffer solution (pH = 6.6) and 5 mL 1% potassium ferricyanide solution were mixed and reacted at 50 °C for 20 min. Then, it was mixed with 5 mL of 10% trichloroacetic acid and left for 10 min. Then, 2.5 mL supernatant was mixed with 2.5 mL distilled water and 0.5 mL of 0.1% ferric chloride solution for 5 min, and the absorbance was measured at a 700 nm wavelength. With Vc as a control, the greater the absorbance under the same condition, the stronger the reduction force. Each group was measured in parallel 3 times.

#### 3.5.2. Determination of Hydroxyl Radical Scavenging Ability

A 1 mL sample solution with different concentrations was mixed with 1 mL of 10 mmol/L ferrous sulfates, 1 mL of 10 mmol/L hydrogen peroxide solution, and 1 mL of 10 mmol/L salicylic acid ethanol solution and left for 20 min. The absorbance A1 was determined at a 510 nm wavelength. The above salicylic acid ethanol solution was replaced with an equal volume of ethanol, and the absorbance A2 was determined at a 510 nm wavelength. The solution to be tested was replaced by 70% methanol of the same volume, and the absorbance A0 was determined at a 510 nm wavelength. Vc was used as the positive control, and each group was measured 3 times in parallel. Hydroxyl radical clearance was calculated according to the following formula:

Hydroxyl radical clearance (%) = [1 − (A1 − A2)/A0] * 100%(1)

#### 3.5.3. Determination of DPPH Free Radical Scavenging Ability

The 0.2 mL sample solution was mixed with 2 mL of 0.1 mmol/L DPPH ethanol solution, and the absorbance A1 was determined at 517 nm after standing for 30 min away from light. The DPPH ethanol solution was replaced with an equal volume of anhydrous ethanol. The absorbance A2 was determined at a 510 nm wavelength. Then, 2 mL DPPH ethanol solution was mixed with 2 mL 70% methanol. The absorbance A0 was determined at 517 nm, Vc was used as the positive control, and the following formula calculated the DPPH clearance rate:

DPPH free radical clearance (%) = [1 − (A1 − A2)/A0] * 100%(2)

### 3.6. Evaluation of Hypolipidemic Activity In Vitro

#### 3.6.1. Cell Culture

HepG2 cells were purchased from the National Infrastructure of Cell Line Resource (Beijing, China) and were cultured with RPMI 1640 in an incubator with 5% CO_2_ at 37 °C.

#### 3.6.2. Cell Viability Assay

The viability of the cells was evaluated with an MTT assay. The cells were seeded into a 96–well plate at 3 × 10^4^ cells/well for 24 h (*n* = 6) and then treated with various concentrations of sodium oleate, simvastatin, and FFN. After 24 h incubation, the absorbance was measured using a microplate reader (Molecular Devices, San Jose, CA, USA) at 570 nm.

#### 3.6.3. Sodium Oleate Induces the Accumulation of TG in HepG2 Cells

HepG2 cells were seeded into 6–well plates at 5 × 10^4^ cells/well for 24 h (*n* = 6) in a humidified 5% CO_2_ incubator at 37 °C and then treated with various concentrations of sodium oleate (10, 20, 40, 60 μg/mL). After 24 h of incubation, the content of TG accumulation was measured by the Triglyceride Assay Kit according to the manufacturer’s instructions. Then, oil red O staining was used to characterize intracellular lipid accumulation using a commercially available oil red O kit following the manufacturer’s protocol.

#### 3.6.4. Measurement of Intracellular TG and TC Accumulation

HepG2 cells were seeded into 6–well plates at 5 × 10^4^ cells/well for 24 h (*n* = 6) in a humidified 5% CO_2_ incubator at 37 °C. Based on the optimal concentration of SO (60 μg/mL) according to experiments as described earlier, cells were induced by SO and cultured with 5% CO_2_ at 37 °C for 24 h. Then, the cell medium containing different concentrations of flavonoid extraction was used for another 24 h. *N. nucifera* samples were selected as the blank control group, using simvastatin (Sim) as the positive control group. Finally, the cells were collected and dissolved in 2% Triton X–100 lysis buffer in PBS for 40 min on ice. The accumulation of TC and TG was measured by the Total Cholesterol Assay Kit and Triglyceride Assay Kit according to the manufacturer’s instructions.

### 3.7. Chromatographic Conditions for Quantitative Fingerprint

HPLC analysis was performed on an ACQUITY^TM^ HPLC system (Waters, Milford, MA, USA). Samples were separated on a Sunfire C18 (4.6 mm × 250 mm, 5 µm) at 25 °C. The mobile phase consisted of methanol (A) and 0.2% formic acid aqueous solution (B), and the elution gradient was set as follows: 0–10 min, 3% A; 10–20 min, 3–20% A; 20–40 min, 20% A; 40–50 min, 20–35% A; 50–65 min, 35% A; 65–75 min, 35–45% A; 75–85 min, 45–80% A; 85–90 min, 80–85% A. The operating parameters were set as follows: the flow rate was 0.8 mL/min; the detection wavelength was 360 nm; the injection volume was 10 µL.

### 3.8. Data Analysis and Chemometric Methods

The fingerprints of *N. nucifera* were established by the Chinese Medicine Chromatographic Fingerprint Similarity Evaluation System software (version 2012) to create the reference chromatographic fingerprints, and the SA data of the common peaks were obtained for different batches of *N. nucifera*. The obtained data generated in the experiment were analyzed via CA, PCA, and OPLS–DA using SPSS (version 24.0) and SIMCA (version 14.1). The graphing software was GraphPad Prism 8.3. One–way analysis of variance (ANOVA) was used for normality, and non–parametric tests were used for non–normality. Data were compared between groups using the LSD method. The results were expressed as mean ± standard deviation (X ± s). *p* < 0.05 indicates a significant difference, *p* < 0.01 indicates a highly significant difference, and *p* < 0.001 indicates a highly significant difference.

## 4. Conclusions

In recent years, studies on medicinal edible plants have increased significantly. As a homologous plant of the hypolipidemic drug, *N. nucifera* is widely distributed. However, there are obvious differences in the chemical components of *N. nucifera* from different sources. In contrast, the differences in the types and contents of chemical components are closely related to their function.

In this study, we used chemical characterization to study the pharmacodynamic material basis in *N. nucifera*, and 105 compounds were identified from *N. nucifera*, which laid a foundation for the subsequent screening of markers. Then, we confirmed the antioxidant effect and hypolipidemic effect of FFN with in vitro experiments. FFN can significantly reduce the TC and TG content in HepG2 cells and has a good antioxidant effect, which can protect vascular endothelial cells and antioxidants and remove excess oxygen free radicals in the body, significantly improving the imbalance between oxidation and antioxidation caused by hyperlipidemia and then regulating dyslipidemia. Since flavonoids have been confirmed as one of the active ingredients in *N. nucifera*, we could study quality control methods for *N. nucifera* by focusing on flavonoids. So, we comprehensively analyzed the quantitative fingerprint of 51 batches of *N. nucifera* and 10 batches of FFN combined with HCA, PCA, and OPLS–DA chemical pattern recognition technology and screened out six flavonoid compounds as markers to evaluate the quality of *N. nucifera*. The results showed that the contents of six flavonoids in *N. nucifera* from different origins were quite different, which may be related to the planting environment, processing, and storage methods. Subsequently, the quality evaluation standard of *N. nucifera* was established with flavonoids as the index, aiming to develop a more systematic and scientific method for evaluating and controlling the quality of *N. nucifera*. PCA, CA, and HCA were used to analyze the relationship between the total content and mass of six flavonoids in *N. nucifera*. The results showed that the ratio of quercetin–3–O–*β*–D–glucuronide content to the total content of six differential components in 82.35% (42 batches) of *N. nucifera* was more than 50%, indicating that the content of quercetin–3–O–*β*–D–glucuronide should be paid attention to in the quality control of *N. nucifera*.

Despite these research results, some limitations remain. Since this study only explored the antioxidant effect and hypolipidemic effect of FFN, the antioxidant effect and hypolipidemic effect of those markers were not studied separately. Therefore, questions like “whether the antioxidant effect and hypolipidemic effect of these markers are significant” or “whether there are interactions between the compounds” were not clear yet. So, we will conduct further research on the hypolipidemic effects of single markers and use network pharmacology and molecular docking technology to dock the markers with the hyperlipidemia targets to further elucidate the hypolipidemic mechanism of flavonoid markers in *N. nucifera* from a chemical and biological perspective.

In summary, the comprehensive use of HPLC fingerprint and chemometrics analysis methods, based on the flavonoid components of lotus leaf quality evaluation suggestions, ensures the clinical efficacy of lotus leaf to provide a scientific basis, but also provides a new idea for the efficacy of traditional Chinese medicine-related comprehensive quality evaluation research. This strategy can be widely used in the analysis and research of traditional Chinese medicine and food resources, which provides a powerful tool for the effective utilization and targeted development of resources.

## Figures and Tables

**Table 1:** The multi–index components of *N. nucifera* determined by UPLC–Q–OF/MS in negative ESI mode.

No.	RT (min)	Chemical Formula	Theoretical *m*/*z*	Measured (*m*/*z*)	Fragment ion (*m*/*z*)	MS/MS (*m*/*z*)	Compound
1	22.69	C_27_H_30_O_16_	609.14661	609.14611	1.599	301.03607, 300.02756, 255.02992, 151.03654	Rutin
2	22.63	C_21_H_20_O_12_	463.08820	463.10275	−0.002	301.07162, 300.02811, 179.01920, 150.04509	Hyperoside
3	22.38	C_21_H_20_O_12_	463.08820	463.08884	1.738	301.03552, 300.02759, 271.05585, 255.00177	Isoquercitrin
4	22.37	C_21_H_18_O_13_	477.06746	477.06746	1.533	301.03558, 273.03201, 255.03014	Quercetin–3–O–*β*–D–glucuronide
5	24.09	C_21_H_20_O_11_	447.09328	447.09363	1.442	285.04044, 257.02994, 227.03503	Astragalin
6	26.23	C_15_H_10_O_7_	301.03538	301.03552	1.241	243.03046, 178.99863, 151.00366	Quercetin

**Table 2:** Method validation of quantitative analysis of six multi–index components.

Compounds	Regression Equation	Linear Range (μg/mL)	R^2^	LOQ (μg/mL)	LOD (μg/mL)	Precision (*n* = 6) RSD (%)	Stability (*n* = 6) RSD (%)	Durability (*n* = 3) RSD (%)	Repeatability (*n* = 6) RSD (%)	Recovery (*n* = 6)	
										Mean (%)	RSD (%)
Rutin	*y* = 21,622,053 *x* − 1134	0.0312~0.2500	0.9999	0.3038	0.9205	0.62	1.78	1.94	1.81	100.72	2.88
Hyperoside	*y* = 21,639,858 *x* − 10,921	0.4820~2.4100	1.0000	1.1717	3.5505	0.51	0.47	1.78	1.12	102.14	2.82
Isoquercitrin	*y* = 25,606,420 *x* + 18,249	0.4600~2.3000	0.9995	5.5860	16.9274	0.49	0.46	2.01	0.98	102.09	2.59
Quercetin–3–O–*β*–D–glucuronide	*y* = 26,125,014.4948 *x* + 40,815.0924	1.0240~5.1200	0.9999	3.4312	10.3975	0.53	0.50	2.13	1.23	101.14	0.98
Astragalin	*y* = 20,802,468 *x* + 8487	0.1240~0.6200	0.9998	0.8362	2.5340	0.58	0.58	1.86	2.82	96.75	0.85
Quercetin	*y* = 87,679,780 *x* − 6871	0.0320~0.1600	0.9997	0.3122	0.9461	0.42	0.90	0.44	2.82	102.39	2.33

## Data Availability

Data are contained within the article or supplementary material.

## Supplementary Materials

The following supporting information can be downloaded at: https://www.mdpi.com/article/10.3390/molecules29235798/s1, Figure S1: Evaluation of cell viability values (*n* = 6); Figure S2: Effect of different concentrations of sodium oleate on TG content in HepG2 cells (n = 6); Figure S3: The results of oil red O staining; Figure S4: The scree plot of the principal component; Figure S5: The dendrogram of 51 batches of *N. nucifera* cluster analysis based on the content of 6 flavonoid components; Figure S6: The dendrogram of 51 batches of *N. nucifera* cluster analysis based on the ratio of quercetin–3–O–*β*–D–glucuronide content to the total content of 6 flavonoid index components; Table S1: UPLC–MS/MS data of compounds identified from *N. nucifera*; Table S2: Similarity evaluation results of 50 batches of *N. nucifera*; Table S3: Similarity evaluation results of 10 batches of the FFN; Table S4: Content of six flavonoid differential components in *N. nucifera*; Table S5: Content of six flavonoid differential components in FFN; Table S6: Principal component eigenvalues and variance contribution rate; Table S7: Initial factor loading matrix; Table S8: Results of a comprehensive evaluation of flavonoids in 51 batches of *N. nucifera*; Table S9: Correlation between the composite score and the total content; Table S10: Ratio of quercetin 3–O–β–D–glucuronide content to the total content of 6 flavonoid index components in 51 batches of *N. nucifera*; Table S11: Information of *N. nucifera* in different places (*n* = 51); Table S12: Information of *N. nucifera* used to prepare FFN in different places (*n* = 10).
