# Supplementary material for: Study on the Chemical Characterization and Hypolipidemic Function of Nelumbo nucifera Based on Its Flavonoid Components
Source: Molecules. 2024 Dec 9;29(23):5798. doi: 10.3390/molecules29235798 (PMC11643375; doi:10.3390/molecules29235798)
Supplement: Supplementary file 1 [file molecules-29-05798-s001.zip › molecules-3344792-supplementary.pdf]

Supplementary Table

Table S1 UPLC-MS/MS data of compounds identified from *N. nucifera*

Table S2 Similarity evaluation results of 50 batches of *N. nucifera*

Table S3 Similarity evaluation results of 10 batches of the FFN

Table S4 Table Content of six flavonoid differential components in *N. nucifera*

Table S5 Table Content of six flavonoid differential components in FFN

Table S6 Principal component eigenvalues and variance contribution rate

Table S7 Initial factor loading matrix

Table S8 Results of a comprehensive evaluation of flavonoids in 51 batches of *N. nucifera*

Table S9 Correlation between the composite score and the total content

Table S10 Ratio of quercetin 3-O-β-D-glucuronide content to the total content of 6 flavonoid index components in 51 batches of *N. nucifera*

Table S11 Information of *N. nucifera* in different places (n = 51)

Table S12 Information of *N. nucifera* used to prepare FFN in different places (n = 10)

Table S1 UPLC-MS/MS data of compounds identified from *N. nucifera*

Number	t_R/min	Elemental composition	Proposed Ions	Theoretical Mass (*m*/*z*)	Experimental Mass (*m*/*z*)	Mass Error (ppm)	MS/MS (*m*/*z*)	Identification
1	0.54	$C_5H_9NO_4$	[M+H]	148.06043	148.06041	-0.024	102.07934、84.06014	glutamic acid
2	0.94	$C_6H_{14}N_2O_2$	[M+H]	147.11280	147.11284	0.036	130.08600、84.06014	lysine
3	0.89	$C_4H_6O_6$	[M-H]	149.00916	149.00903	0.966	87.00887、72.99299	tartaric acid
4	1.06	$C_5H_{11}NO_2$	[M+H]	118.08626	118.08652	0.265	72.08139、59.07369	valine
5	1.11	$C_6H_{12}O_7$	[M-H]	195.05103	195.05095	1.051	159.02974、129.01936、75.00871	gluconic acid
6	1.13	$C_5H_9NO_2$	[M+H]	116.07061	116.07091	0.305	70.06578	proline
7	1.23	$C_{15}H_8O_9$	[M-H]	341.10893	341.10886	1.022	179.05605、101.02435、89.02442	caffeic acid glucoside
8	1.43	$C_4H_4O_4$	[M-H]	115.00368	115.00365	1.065	71.01385	butene dioic acid
9	1.63	$C_4H_6O_5$	[M-H]	133.01425	133.01419	1.040	115.00364、71.01382	malic acid
10	1.77	$C_6H_6N_2O$	[M+H]	123.05529	123.05550	0.211	96.04472、80.04997	nicotinamide
11	1.94	$C_9H_{12}NO_2$	[M+H]	167.09408	167.09084	-3.420	121.06477、120.08089、103.05429	phenylalanine

12	2.32	$C_6H_8O_7$	[M-H]	191.01973	191.01978	1.101	129.01927、111.00877、87.00875	citric acid
13	2.62	$C_4H_6O_4$	[M-H]	117.01933	117.01934	1.105	73.02945	succinic acid
14	2.69	$C_4H_4O_4$	[M-H]	115.00368	115.00364	1.055	71.01384	butene dioic acid
15	3.21	$C_6H_{13}NO_2$	[M+H]	132.10191	132.10202	0.115	86.09686	isoleucine
16	3.41	$C_6H_{13}NO_2$	[M+H]	132.10191	132.10210	0.195	86.09691	leucin
17	3.54	$C_9H_{11}NO_3$	[M+H]	182.08117	182.08125	0.080	165.05443、147.04387、136.07555、91.05463	tyrosine
18	3.54	$C_9H_{11}NO_3$	[M+H]	182.08117	182.08125	0.080	165.05441、147.04378、136.07555、91.05463	tyrosine
19	4.35	$C_{10}H_{13}N_5O_4$	[M+H]	268.10403	268.10403	0.000	136.06168	adenosine
20	8.84	$C_7H_6O_4$	[M-H]	153.01933	153.01939	1.115	109.02952	protocatechuic acid
21	9.28	$C_8H_{10}O_3$	[M-H]	153.05572	153.05579	1.169	123.04516、109.02945	3,4-dihydroxyphenylethanol
22	11.35	$C_7H_6O_3$	[M-H]	137.02442	137.02440	1.079	109.02957	4-hydroxybenzoic acid
23	12.11	$C_7H_{14}O_4$	[M+H]	163.09649	163.09667	1.070	117.05494、115.95451	homomevalonic acid
24	12.28	$C_{11}H_{12}N_2O_2$	[M+H]	205.09715	205.09688	-0.274	188.07045、146.05992	tryptophan
25	13.56	$C_{16}H_{17}NO_3$	[M+H]	272.12812	272.12805	-0.070	255.10132、239.14879、209.15393、164.98456、161.02950、142.94812	norcoclaurine

26	14.27	$C_{15}H_{14}O_6$	[M+H]	291.08631	291.08572	-0.595	207.06487、165.05446、139.03883	catechin/epicatechin
27	14.47	$C_{15}H_{14}O_6$	[M+H]	291.08631	291.08554	-0.775	207.06491、165.05440、139.03879	catechin/epicatechin
28	14.48	$C_{18}H_{14}O_8$	[M-H]	357.06159	357.05960	-2.174	311.05396、122.03725、77.95902、68.02304、52.06055	quercetin-3-propyl ester
29	15.26	$C_{30}H_{26}O_{12}$	[M-H]	577.13515	577.13580	1.747	451.10291、425.08813、407.07788、289.07202、287.05643、245.08209、161.02444、125.02439	procyanidin dimmer
30	15.45	$C_9H_6O_4$	[M+H]	179.03389	179.03389	0.027	149.01222、132.95839、104.96371	dihydroxy coumarin
31	15.47	$C_{30}H_{26}O_{12}$	[M-H]	577.13515	577.13568	1.627	425.08813、407.077718、289.07187、245.08214、125.024433	procyanidin dimmer
32	16.05	$C_{17}H_{19}NO_3$	[M+H]	286.14377	286.14371	-0.060	269.11719、239.22469、209.15372、177.00708、174.07611	coclaurine
33	16.07	$C_9H_8O_4$	[M-H]	179.03498	179.03500	1.115	162.93985、145.05060、116.92860	caffeic acid
34	16.22	$C_{18}H_{21}NO_3$	[M+H]	300.15942	300.15933	-0.090	269.11707、239.22507、209.15367、192.97919、174.07614、143.03394	N-methylisococlaurine
35	16.42	$C_{18}H_{21}NO_3$	[M+H]	300.15942	300.15930	-0.120	269.11710、239.22472、209.15370、192.12299、174.07619、146.08119	N-methylcoclaurine
36	16.86	$C_{37}H_{42}N_2O_6$	[M+H]	611.31156	611.31152	-0.043	567.17389、485.24228、120.96634	liensinine
37	17.70	$C_{19}H_{23}NO_3$	[M+H]	314.17507	314.17505	-0.020	283.13129、251.24878、206.10239	armepavine
38	17.73	$C_{13}H_{20}O_2$	[M+H]	209.15361	209.15364	0.034	191.14256、177.00670、167.14273、151.11153、133.10098、121.10120	(E)-3-oxo-retro-α-ionol

39	18.37	$C_{18}H_{21}NO_3$	[M+H]	300.15942	300.15933	-0.090	283.13284、252.08681、239.22520、209.15375、192.12300、188.09167	N-norarmepavine
40	18.71	$C_{38}H_{44}N_2O_6$	[M+H]	625.32721	625.32727	0.056	580.07922、502.82541、120.96632	neferine
41	18.73	$C_9H_{18}O_3$	[M-H]	163.04007	163.04016	1.189	119.05023	p-coumaroylagmatine
42	18.79	$C_{21}H_{22}O_{11}$	[M-H]	449.10893	449.10953	1.692	287.05637、259.06122、125.02438	taxifolin 7-rhamnoside
43	18.92	$C_{38}H_{44}N_2O_6$	[M+H]	625.32721	625.32690	-0.314	489.23746、206.11731、121.06481	dauricine
44	19.28	$C_{18}H_{19}NO_2$	[M+H]	282.14886	282.14880	-0.055	251.10654、236.11284、208.14139	O-nornuciferine
45	19.85	$C_{17}H_{17}NO_2$	[M+H]	268.13321	286.14371	-0.060	269.11508、252.10956、237.08936、252.10956、220.08357、209.09557、192.08861、175.07512、143.04900	asimilobine
46	19.93	$C_{10}H_{10}O_4$	[M-H]	193.05063	193.05067	1.135	149.02435、121.02953	ferulic acid
47	19.95	$C_{17}H_{19}NO_3$	[M+H]	286.14377	286.14371	-0.060	269.11722、252.10979、239.22469、209.03317	N-demethylcoclaurine
48	20.72	$C_{26}H_{28}O_{16}$	[M-H]	595.13046	595.13080	1.439	301.03583、300.02768、271.02496	quercetin-3-O-pentosylhexoside
49	20.79	$C_{21}H_{20}O_{13}$	[M-H]	479.08311	479.08362	1.603	317.03046、316.02252、287.01993、271.02496	myricetin 3-O-galactoside
50	20.88	$C_{26}H_{27}O_{16}$	[M-H]	594.12263	594.12238	0.844	301.03540、300.02759	quercetin 3-O-arabinose-(1→2)-galactoside
51	20.91	$C_{26}H_{28}O_{16}$	[M-H]	595.13046	595.13129	1.929	301.03552、300.02759、271.02481、255.02995、243.03001、178.99860、151.00366	quercetin-3-O-β-D-xylopyranosyl(1→2)-β-D-glucopyranoside

52	20.99	$C_{21}H_{20}O_{13}$	[M-H]	479.08311	479.08337	1.353	317.02994、316.02243、287.01974、271.02472	myricetin 3-β-D-glucopyranoside
53	21.41	$C_7H_6O_5$	[M-H]	169.01425	169.01422	1.070	125.02430、124.04038	gallic acid
54	22.14	$C_{19}H_{21}NO_2$	[M+H]	296.16451	296.16443	0.045	265.12177、250.09831、234.10344、219.08005、219.08005	N-noratherosperminine
55	22.27	$C_{26}H_{28}O_{15}$	[M-H]	579.13554	579.13605	1.604	285.04065、284.03290、255.03008、227.03517	leucoside
56	22.34	$C_{19}H_{21}NO_2$	[M+H]	296.16451	296.16443	-0.075	265.12225、250.09792、234.10376	nuciferine
57	22.37	$C_{21}H_{18}O_{13}$	[M-H]	477.06746	477.06746	1.533	301.03558、273.03201、255.03014	quercetin-3-O-glucuronide
58	22.38	$C_{21}H_{20}O_{12}$	[M-H]	463.08820	463.08884	1.738	301.03552、300.02759、271.05585、255.00177	isoquercitrin
59	22.42	$C_{21}H_{20}O_{12}$	[M-H]	463.08820	463.08871	1.608	301.03549、300.02737、272.54156	quercetin-3-O-hexoside
60	22.63	$C_{21}H_{20}O_{12}$	[M-H]	463.08820	463.10275	-0.002	301.07162、300.02811、179.01920、150.04509	hyperoside
61	22.69	$C_{27}H_{30}O_{16}$	[M-H]	609.14661	609.14611	1.599	301.03607、300.02756、255.02992、151.03654	rutin
62	22.86	$C_{20}H_{18}O_{11}$	[M-H]	433.07763	433.08871	1.608	301.03549、300.02771、271.02490、255.02994	quercetin 3-O-beta-D-xylopyranoside
63	22.93	$C_{18}H_{17}NO_2$	[M+H]	280.13321	280.13315	-0.055	249.09111、220.11797、191.14297	roemerine
64	23.00	$C_{18}H_{19}NO_2$	[M+H]	282.14886	282.14877	-0.085	265.12219、250.09898、235.07483、207.13770	N-nornuciferine

65	23.50	$C_{17}H_{15}NO_2$	[M+H]	266.11756	266.11749	-0.065	249.09087、219.08052、191.08554	anonaine
66	23.63	$C_{22}H_{22}O_{11}$	[M+H]	463.12349	463.08701	-0.138	301.07007、286.04660、258.05167	homoplantaginin
67	23.69	$C_{21}H_{20}O_{11}$	[M-H]	447.09328	447.06772	1.353	285.03546、267.02591、151.00363	cynaroside
68	23.70	$C_{21}H_{20}O_{11}$	[M-H]	447.10784	447.10760	1.502	285.04092、284.03271、255.02992、227.03506	kaempferol-3-O-hexoside
69	23.77	$C_9H_{16}O_4$	[M-H]	187.09758	187.09763	1.145	169.01424、125.09718、123.00883	azelaic acid
70	23.79	$C_{21}H_{18}O_{12}$	[M-H]	461.07255	461.07217	0.718	285.04050、284.12836、255.21916、227.12527	kaempferol-3-O-glucuronide
71	23.80	$C_{22}H_{22}O_{11}$	[M+H]	463.12349	463.12292	-0.568	301.07004、286.04660	diosmetin-7-O-β-D-glucopyranoside
72	23.90	$C_{21}H_{20}O_{11}$	[M-H]	447.09328	449.10776	1.442	301.03510、300.02759、271.02502、255.02965	quercetin-3-O-rhamnoside
73	23.99	$C_{19}H_{21}NO_3$	[M+H]	312.15942	312.15927	-0.150	283.15189、266.11719、255.21899、251.10689、225.14827	pronuciferine
74	24.00	$C_{27}H_{30}O_{15}$	[M-H]	593.15119	593.15167	1.574	285.04047、284.03259、255.02994、227.03503	kaempferol-3-O-rutinoside
75	24.06	$C_{22}H_{22}O_{12}$	[M-H]	477.11840	477.11838	-0.022	315.14169、314.16586、297.17691、271.81812、242.09988	rhamnetin-3-O-β-D-glucopyranoside
76	24.07	$C_{15}H_{12}O_6$	[M-H]	287.05611	287.05618	1.165	269.06302	eriodictyol
77	24.09	$C_{21}H_{20}O_{11}$	[M-H]	447.09328	447.09363	1.442	285.04044、257.02994、227.03503	astragalin
78	24.17	$C_{23}H_{24}O_{13}$	[M-H]	507.11441	507.11496	1.643	345.06183、344.05386、329.03021	syringetin-3-O-glucoside

79	24.29	$C_{22}H_{20}O_{13}$	[M-H]	491.08311	491.08105	-0.967	315.05142、314.16553、301.02771、299.01831、271.02493、165.01926	isorhamnetin-3-O-glucuronide
80	24.30	$C_{28}H_{32}O_{16}$	[M-H]	623.16176	623.16235	1.689	315.05124、314.04340、301.02771、299.01993、271.02484、243.02962	isorhamnetin-3-O-α-L-rhamnopyranosyl-(1→6)-β-D-glucopyranoside
81	24.69	$C_{17}H_9NO_3$	[M+H]	276.06552	276.06540	-0.120	249.09103	liriodenine
82	24.94	$C_{27}H_{30}O_{16}$	[M-H]	609.14611	609.14685	1.839	301.01978、300.02805、271.02484、255.03041	quercetin 3-O-rutinoside
83	25.02	$C_{27}H_{30}O_{16}$	[M-H]	609.14611	609.14685	0.109	315.05084、314.04343、301.02805、299.01978、271.02484	isorhamnetin-3-O-pentosylhexoside
84	26.04	$C_{15}H_{10}O_4$	[M-H]	253.05063	253.05087	1.335	223.94328、222.94629、208.02986	dihydroxy flavone
85	26.16	$C_{28}H_{32}O_{16}$	[M-H]	623.16176	623.16211	1.449	315.05139、314.04343、300.02777、299.02008	narcissoside
86	26.23	$C_{15}H_{10}O_7$	[M-H]	301.03538	301.03552	1.241	271.04074、255.02985、243.03046、178.99863、151.00366	quercetin
87	26.43	$C_{16}H_{12}O_7$	[M-H]	315.05103	315.05139	1.461	300.02774、271.02496、243.03000、151.00366	isorhamnetin
88	26.44	$C_{16}H_{12}O_7$	[M-H]	315.05103	315.05142	1.491	300.02777、246.89478、216.92859、165.01932	eupafolin
89	26.55	$C_{22}H_{22}O_{12}$	[M-H]	477.10385	477.10443	1.678	315.05154、314.04349、301.03571、299.01993、259.06033、243.03073、165.01907	isorhamnetin-3-O-glucoside
90	26.81	$C_{15}H_{10}O_6$	[M-H]	285.04046	285.04062	1.256	266.95714、257.02163、241.01382、217.01749、199.02311、175.01884、151.00360、133.02939	luteolin

91	27.94	$C_{15}H_{10}O_6$	[M-H]	285.04046	285.04053	1.166	257.02844、227.03473	kaempferol
92	28.17	$C_{18}H_{13}NO_3$	[M+H]	292.09682	292.09665	-0.170	277.06815、265.17712、249.14626、246.08165	lysicamine
93	28.37	$C_{16}H_{12}O_6$	[M-H]	299.05611	299.05609	1.075	287.03268、286.04648、283.02472、269.03778、255.03026、241.03038、177.03969、153.00383	diosmetin
94	29.11	$C_{15}H_{22}O$	[M+H]	219.17434	219.17435	0.008	203.14279、174.11253、121.10116	aristolone
95	30.24	$C_{19}H_{19}NO_3$	[M+H]	310.14377	310.14355	-0.180	278.11673、265.12170、250.12225、233.09563、218.07254	N-formylnornuciferine
96	31.59	$C_{16}H_{12}O_5$	[M-H]	283.06120	283.06128	1.180	268.03781	oroxylin A
97	31.74	$C_{16}H_{12}O_5$	[M+H]	285.07575	285.07578	0.030	270.08798、252.07860、179.05428	wogonin
98	31.76	$C_{16}H_{14}O_4$	[M-H]	269.08193	269.08203	1.195	148.01653、119.05014、91.01881	imperatorin
99	33.50	$C_{18}H_{32}O_5$	[M+H]	329.23225	329.22983	-2.421	309.20578、291.19519、239.22479、228.97849、220.93480、212.11835、183.97601、171.06264、139.05032	9,12,13-trihydroxyoctadecadienoic acid
100	34.43	$C_{15}H_{20}O$	[M+H]	217.15869	217.15900	0.308	119.08559、91.05460	(+)-ar-Turmerone
101	35.09	$C_{19}H_{19}NO_2$	[M+H]	294.14886	294.14874	-0.115	279.15897	dehydronuciferine
102	38.83	$C_{10}H_{10}O_3$	[M+H]	179.07027	179.06787	-2.401	161.05951、133.06465、79.05472	4-methoxycinnamic acid
103	38.92	$C_{18}H_{34}O_5$	[M+H]	331.24790	331.24826	0.359	311.25748、293.17487、226.95149、211.10513、183.12399、171.06276、	9,12,13-trihydroxyoctadecenoic acid

							139.05022、127.03477	
104	40.03	$C_{18}H_{32}O_2$	[M-H]	279.23295	279.23288	1.023	69.16511	linoleic acid
105	41.67	$C_{18}H_{36}O_2$	[M-H]	283.26425	283.26419	1.003	253.92659	stearic acid

*Comparison with standards

Table S2 Similarity evaluation results of 50 batches of *N. nucifera*

Samples	Similarity	Samples	Similarity	Samples	Similarity
S1	0.989	S18	0.840	S35	0.987
S2	0.995	S19	0.987	S36	0.994
S3	1.000	S20	0.986	S37	0.989
S4	0.922	S21	0.987	S38	0.986
S5	0.993	S22	0.988	S39	0.990
S6	0.990	S23	0.986	S40	0.983
S7	0.994	S24	0.988	S41	0.988
S8	0.983	S25	0.988	S42	0.857
S9	0.985	S26	0.996	S43	0.993
S10	0.929	S27	0.984	S44	0.999
S11	0.912	S28	0.998	S45	0.797
S12	0.942	S29	0.986	S46	0.830
S13	0.987	S30	0.988	S47	0.856
S14	0.986	S31	0.999	S48	0.989
S15	0.999	S32	0.988	S49	0.988
S16	0.998	S33	0.999	S50	0.987
S17	0.815	S34	0.988	S51	0.986

Table S3 Similarity evaluation results of 10 batches of the flavonoid fraction of *N. nucifera*

Samples	Similarity	Samples	Similarity
S1	0.796	S6	0.996
S2	0.996	S7	0.999
S3	0.994	S8	0.998
S4	0.999	S9	0.996
S5	0.996	S10	0.995

Table S4 Content of six flavonoid differential components in *N. nucifera*

Number	Content（mg/g）						
	Rutin	Hyperoside	Isoquercitrin	Quercetin 3-O-β-D-Glucuronide	Astragalin	Quercetin	Totol

1	0.1459	2.9919	3.6063	8.6550	1.0234	0.2039	16.6264
2	0.1216	1.9939	2.7042	6.8833	0.5805	0.1911	12.4747
3	0.1484	1.2902	2.8636	7.0472	0.4502	0.1891	11.9886
4	0.1410	0.3892	3.2970	6.9583	0.2013	0.3056	11.2924
5	0.1426	1.0302	2.8095	8.9369	0.2720	0.2139	13.4051
6	0.0967	1.4702	2.9182	5.2968	0.5569	0.1707	10.5094
7	0.0674	0.4106	1.8744	4.7032	0.1122	0.0634	7.2312
8	0.2469	0.4273	3.6124	11.3762	0.2378	0.1355	16.0360
9	0.1198	2.5600	2.8343	6.8511	0.7219	0.1809	13.2679
10	0.1179	3.9254	3.3902	6.1734	1.2320	0.1755	15.0144
11	0.1126	5.5595	4.1952	8.0464	1.9885	0.3942	20.2964
12	0.0949	3.2075	2.5210	5.4923	0.7550	0.1329	12.2035
13	0.1110	0.4547	2.8330	8.5790	0.1308	0.1236	12.2322
14	0.0762	0.3247	2.3622	7.0479	0.1044	0.0715	9.9868
15	0.1178	1.7353	2.8618	7.5235	0.4275	0.1207	12.7867
16	0.1373	2.0164	3.3459	7.8940	0.7100	0.1700	14.2736
17	0.0355	2.6449	1.5147	2.4584	0.6733	0.2860	7.6129
18	0.0744	4.5740	2.7484	4.6460	1.3006	0.1304	13.4738
19	0.1524	0.3658	3.0357	8.4559	0.1706	0.0148	12.1952
20	0.0849	0.5098	3.0129	9.5916	0.1744	0.0113	13.3849
21	0.1690	0.3806	3.2256	7.5347	0.1656	0.2534	11.7289
22	0.0768	0.2419	1.9243	4.9721	0.0859	0.0567	7.3577
23	0.0964	0.0346	2.5940	7.5960	0.1265	0.1072	10.5547
24	0.0944	0.4151	2.9625	8.2470	0.1448	0.1105	11.9742
25	0.0930	0.3547	2.6707	7.2944	0.1366	0.0971	10.6466
26	0.1086	1.9768	2.8522	7.1989	0.4993	0.1980	12.8338
27	0.1689	0.4552	3.5504	11.0311	0.2165	0.0736	15.4959
28	0.1187	1.4016	2.7335	7.8767	0.3633	0.1337	12.6275
29	0.1648	0.4192	3.4111	9.9313	0.1918	0.1330	14.2511
30	0.1817	0.5520	4.0844	11.4653	0.2281	0.0877	16.5991
31	0.1555	1.6891	3.7651	8.2817	0.6072	0.1537	14.6523
32	0.1641	0.4254	3.7548	9.6468	0.2350	0.0780	14.3041
33	0.1741	2.0050	3.4458	9.4417	0.5714	0.0734	15.7114
34	0.1734	0.5367	4.3521	9.3552	0.2666	0.0944	14.7784
35	0.1241	0.3184	3.0914	7.3705	0.1796	0.0109	11.0949
36	0.0977	0.5805	2.6932	6.4988	0.2064	0.0951	10.1717
37	0.1394	0.4318	3.4832	8.4856	0.1904	0.0751	12.8055

38	0.1100	0.3029	2.5256	7.4566	0.1422	0.0943	10.6317
39	0.1264	0.5036	3.5084	8.5033	0.2048	0.1335	12.9800
40	0.0657	0.1919	1.5059	4.8106	0.0855	0.1706	6.8302
41	0.1369	0.4705	4.4790	10.8547	0.2118	0.1315	16.2844
42	0.0661	3.4393	2.4157	3.7480	1.1296	0.1196	10.9183
43	0.1626	0.7905	3.8776	9.7703	0.3332	0.0882	15.0222
44	0.1335	1.4194	3.1373	8.5148	0.4686	0.1173	13.7910
45	0.0601	5.0929	3.2607	4.2877	1.5287	0.1501	14.3803
46	0.0827	4.8760	3.2089	4.7114	1.5169	0.1993	14.5952
47	0.1199	5.1751	3.0833	5.6544	1.4434	0.1592	15.6353
48	0.1346	0.4186	3.0302	8.1114	0.1657	0.1347	11.9952
49	0.1203	0.3821	3.3522	8.4268	0.1928	0.0958	12.5700
50	0.1373	0.3483	2.9390	8.1428	0.1761	0.0848	11.8283
51	0.1331	0.4116	3.1768	9.3717	0.1926	0.1876	13.4734

Table S5 Content of six flavonoid differential components in FFN

Number	Content（mg/g）						
	Rutin	Hyperoside	Isoquercitrin	Quercetin-3-O-β-D-Glucuronide	Astragalin	Quercetin	Totol
1	0.1872	6.5770	3.7924	7.3575	3.2773	0.3079	21.4993
2	0.4447	3.4572	8.8429	28.8278	0.8807	1.8320	44.2853
3	0.6765	1.0562	9.3935	36.9243	1.0056	0.4047	49.4608
4	0.4230	3.0306	8.2353	30.9902	1.5279	0.3988	44.6058
5	0.1860	0.5259	4.2013	13.9111	0.5264	0.2460	19.5967
6	0.2158	0.8050	5.5600	19.5123	0.5537	0.3064	26.9532
7	0.1141	1.7799	3.6402	12.9832	1.0942	0.2109	19.8225
8	0.2380	2.8480	5.0750	17.0482	1.2173	0.2830	26.7095
9	0.1428	1.5541	3.4379	10.5662	1.0103	0.2295	16.9408
10	0.1816	0.4618	3.6374	13.8455	0.4321	0.1804	18.7388

Table S6 Principal component eigenvalues and variance contribution rate

Principal component	Initial Eigenvalues			Extraction Sums of Squared Loadings		
	Total	Variance (%)	Cumulation (%)	Total	Variance (%)	Cumulation (%)
1	3.032	50.540	50.540	3.032	50.540	50.540
2	1.976	32.938	83.478	1.976	32.938	83.478
3	0.662	11.031	94.509			
4	0.213	3.556	98.066			
5	0.106	1.761	99.827			
6	0.010	0.173	100.000			

Table S7 Initial factor loading matrix

	Principal components	
	Z1	Z2
X1	0.753	0.536
X2	-0.800	0.532
X3	0.477	0.811
X4	0.884	0.379
X5	-0.755	0.608
X6	-0.497	0.484

Table S8 Results of a comprehensive evaluation of flavonoid in 51 batches of *N. nucifera*

Samples	Z1	Z2	Z	Samples	Z1	Z2	Z
S1	0.3475	0.6117	0.3910	S27	0.6230	0.5382	0.5171
S2	0.2996	0.4536	0.3128	S28	0.3889	0.4483	0.3598
S3	0.3510	0.4359	0.3350	S29	0.5645	0.5000	0.4726
S4	0.4057	0.4174	0.3588	S30	0.6534	0.5859	0.5494
S5	0.4644	0.4669	0.4071	S31	0.4122	0.5422	0.4034
S6	0.2488	0.4006	0.2677	S32	0.5580	0.5123	0.4731
S7	0.2626	0.2602	0.2289	S33	0.4534	0.5634	0.4329
S8	0.6440	0.5561	0.5344	S34	0.5513	0.5454	0.4803
S9	0.2692	0.4873	0.3073	S35	0.4334	0.4023	0.3689

S10	0.1638	0.5745	0.2786	S36	0.3626	0.3687	0.3192
S11	0.1646	0.7734	0.3445	S37	0.4929	0.4625	0.4212
S12	0.1616	0.4559	0.2383	S38	0.4232	0.3721	0.3534
S13	0.4805	0.4265	0.4025	S39	0.4882	0.4693	0.4208
S14	0.3982	0.3487	0.3320	S40	0.2670	0.2362	0.2234
S15	0.3563	0.4610	0.3462	S41	0.6335	0.5882	0.5392
S16	0.3614	0.5242	0.3698	S42	0.0426	0.4262	0.1636
S17	0.0051	0.2941	0.0996	S43	0.5459	0.5411	0.4760
S18	0.0370	0.5205	0.1916	S44	0.4270	0.4940	0.3956
S19	0.4865	0.4310	0.4073	S45	-0.0034	0.5701	0.1859
S20	0.5341	0.4633	0.4439	S46	0.0268	0.5724	0.2032
S21	0.4379	0.4263	0.3792	S47	0.0639	0.5987	0.2320
S22	0.2870	0.2630	0.2431	S48	0.4624	0.4266	0.3927
S23	0.4439	0.3689	0.3636	S49	0.4874	0.4516	0.4146
S24	0.4677	0.4229	0.3944	S50	0.4661	0.4184	0.3920
S25	0.4155	0.3773	0.3509	S51	0.5277	0.4718	0.4432
S26	0.3228	0.4662	0.3296				

Table S9 Correlation between the composite score and the total content

		Total content	Composite score
Total content	Correlation coefficient	1.000	0.859**
	Sig.	0.0	0.000
	N	51	51
Composite score	Correlation coefficient	0.859**	1.000
	Sig.	0.000	0.0
	N	51	51

Table S10 Ratio of Quercetin-3-O-β-D-Glucuronide content to total content of 6 flavonoid index components in 51 batches of *N. nucifera*

Samples	Ratio/%	Samples	Ratio/%	Samples	Ratio/%
S1	52.06	S18	32.29	S35	63.30
S2	55.18	S19	34.48	S36	66.43
S3	58.78	S20	69.34	S37	63.89
S4	61.62	S21	71.66	S38	66.27
S5	66.67	S22	64.24	S39	70.14
S6	50.40	S23	67.58	S40	65.51

S7	65.04	S24	71.97	S41	70.43
S8	70.94	S25	68.87	S42	66.66
S9	51.64	S26	68.51	S43	34.33
S10	41.12	S27	56.09	S44	65.04
S11	39.64	S28	71.19	S45	61.74
S12	45.01	S29	62.38	S46	29.82
S13	70.13	S30	69.69	S47	32.28
S14	70.57	S31	69.07	S48	36.16
S15	58.84	S32	56.52	S49	67.62
S16	55.30	S33	67.44	S50	67.04
S17	52.06	S34	60.09	S51	68.84

Table S11 Information of *N. nucifera* in different places (n = 51)

Samples	Origin	Number	Sample	Origin	Number
S1	An Hui	20170201	S27	Jiang Xi	20210501
S2	An Hui	211129	S28	Jiang Xi	210401
S3	An Hui	211201	S29	Shan Dong	200501
S4	An Hui	211215	S30	Shan Dong	200608
S5	An Hui	2019120421	S31	Shan Dong	200805CP254
S6	An Hui	2021030815	S32	Shan Dong	209501
S7	An Hui	211201	S33	Shan Dong	210102CP254
S8	He Bei	201201	S34	Shan Dong	210601
S9	He Bei	201211	S35	Shan Dong	210609
S10	He Bei	201260591	S36	Shan Dong	211201
S11	He Bei	211020	S37	Shan Dong	211206
S12	He Bei	191101	S38	Shan Dong	211210
S13	Hu Bei	211106	S39	Shan Dong	211216
S14	Hu Bei	211205	S40	Shan Dong	211219
S15	Hu Bei	A210203	S41	Shan Dong	145200701
S16	Hu Nan	190801	S42	Shan Dong	Z2110001
S17	Hu Nan	191001	S43	Shan Dong	211101
S18	Hu Nan	200901	S44	Si Chuan	201101
S19	Hu Nan	201201	S45	Si Chuan	200825
S20	Ji Lin	211018	S46	Si Chuan	210514
S21	Ji Lin	211118	S47	Hu Nan	20200501

S22	Jiang Su	210713	S48	Chang Baishan	211018
S23	Jiang Su	211120	S49	Chang Baishan	211204
S24	Jiang Su	211201	S50	Zhe Jiang	211029
S25	Jiang Su	211208	S51	Zhe Jiang	211004
S26	Jiang Xi	20201201			

Table S12 Information of *N. nucifera* used to prepare FFN in different places (n = 10)

Samples	Origin	Number	Samples	Origin	Number
S1	Hu Nan	20170201	S6	Jiang Su	211029
S2	An Hui	2019120421	S7	Jiang Xi	210401
S3	He Bei	211120	S8	Shan Dong	210102CP254
S4	Hu Bei	A210203	S9	Si Chuan	201101
S5	Ji Lin	211018	S10	Zhe Jiang	211129

Supplementary Figure

Figure S1 Evaluation of cell viability values (n = 6)

Figure S2 Effect of Different Concentrations of Sodium Oleate on TG Content in HepG2 Cells (n = 6)

Figure S3 The results of oil red O staining

Figure S4 The Scree plot of the Principal component

Figure S5 The dendrogram of 51 batches of *N. nucifera* cluster analysis based on the content of 6 flavonoid components

Figure S6 The dendrogram of 51 batches of *N. nucifera* cluster analysis based on the ratio of Quercetin-3-O-β-D-Glucuronide content to the total content of 6 flavonoid index components

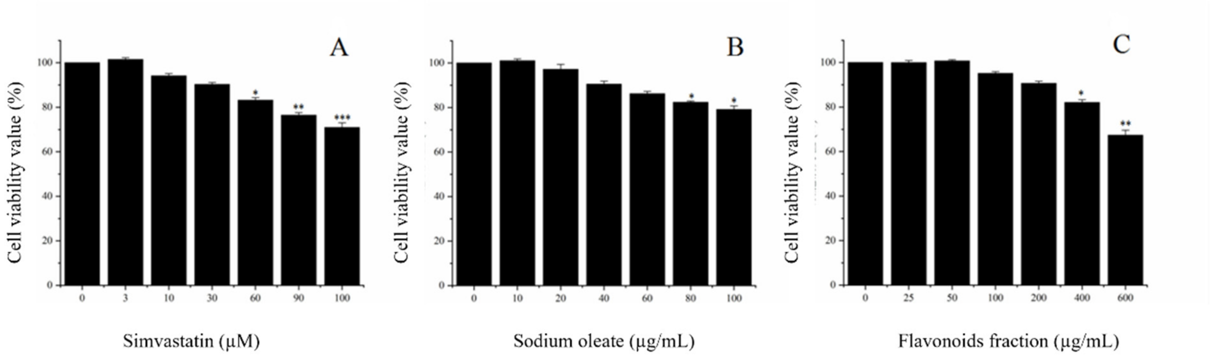

Figure S1 Evaluation of cell viability values (n = 6)

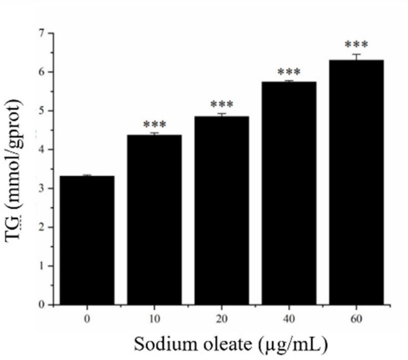

Figure S2 Effect of Different Concentrations of Sodium Oleate on TG Content in HepG2 Cells (n = 6). Compared to KG, *P < 0.05, **P < 0.01, ***P < 0.001

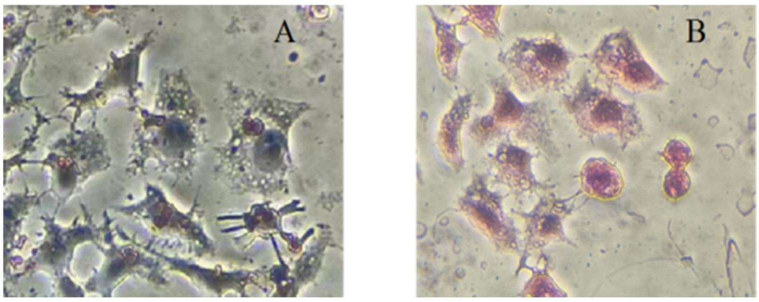

Figure S3 The results of oil red O staining

(blank group (A); sodium oleate group (B))

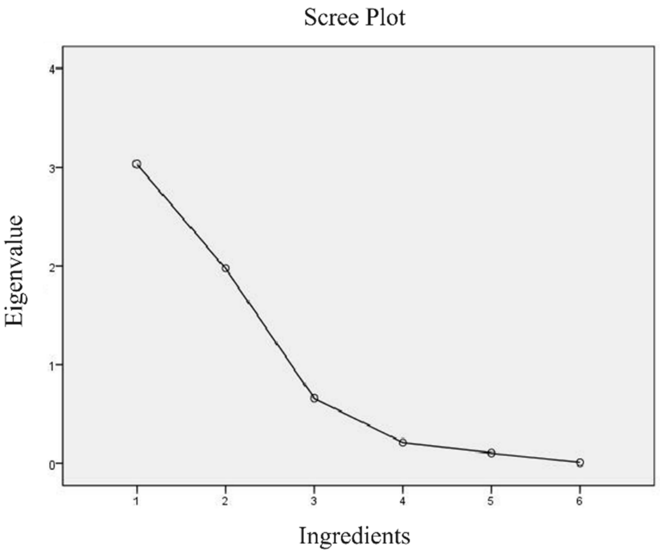

Figure S4 The Scree plot of Principal component

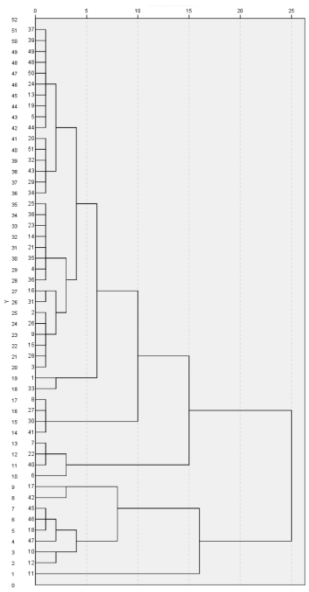

Figure S5 The dendrogram of 51 batches of *N. nucifera* cluster analysis based on the content of 6 flavonoid componrnts

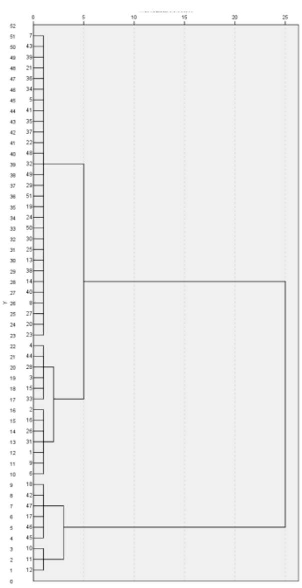

Figure S6 The dendrogram of 51 batches of *N. nucifera* cluster analysis based on the ratio of Quercetin-3-O-*β*-D-Glucuronide content to the total content of 6 flavonoid index components
